# Exploring feasibility and acceptability of an integrated urban gardens and peer nutritional counselling intervention for people with HIV in the Dominican Republic

**DOI:** 10.1017/S1368980023002264

**Published:** 2023-12

**Authors:** Alane Celeste-Villalvir, Amarilis Then-Paulino, Gabriela Armenta, Gipsy Jimenez-Paulino, Kartika Palar, Deshira D Wallace, Kathryn P Derose

**Affiliations:** 1 University of Massachusetts Amherst, Department of Health Promotion and Policy, Amherst, MA 01003, USA; 2 Universidad Autónoma de Santo Domingo, Facultad de Ciencias de la Salud, Av. Alma Mater, Santo Domingo, Dominican Republic; 3 RAND Corporation and Pardee RAND Graduate School, Santa Monica, CA 90401, USA; 4 University of California, San Francisco, Department of Medicine, San Francisco, CA 94143, USA; 5 University of North Carolina at Chapel Hill, Department of Health Behavior, Chapel Hill, NC 27599, USA

**Keywords:** HIV, Food insecurity, Urban gardening, Nutrition, Process evaluation, Dominican Republic

## Abstract

**Objective:**

Food security interventions with people living with HIV (PLHIV) are needed to improve HIV outcomes. This process evaluation of a pilot intervention involving urban gardening and peer nutritional counselling with PLHIV assesses feasibility, acceptability and implementation challenges to inform scale-up.

**Design:**

Mixed methods were used, including quantitative data on intervention participation and feasibility and acceptability among participants (*n* 45) and qualitative data from a purposive sample of participants (*n* 21). Audio-recorded interviews were transcribed and coded using a codebook developed iteratively.

**Setting:**

An HIV clinic in the northwest-central part of the Dominican Republic.

**Results:**

The intervention was feasible for most participants: 84 % attended a garden workshop and 71 % established an urban garden; 91 % received all three core nutritional counselling sessions; and 73 % attended the cooking workshop. The intervention was also highly acceptable: nearly, all participants (93–96 %) rated the gardening as ‘helpful’ or ‘very helpful’ for taking HIV medications, their mental/emotional well-being and staying healthy; similarly, high percentages (89–97 %) rated the nutrition counselling ‘helpful’ or ‘very helpful’ for following a healthy diet, reducing unhealthy foods and increasing fruit/vegetable intake. Garden barriers included lack of space and animals/pests. Transportation barriers impeded nutritional counselling. Harvested veggies were consumed by participants’ households, shared with neighbours and family, and sold in the community. Many emphasised that comradery with other PLHIV helped them cope with HIV-related marginalisation.

**Conclusion:**

An urban gardens and peer nutritional counselling intervention with PLHIV was feasible and acceptable; however, addressing issues of transportation, pests and space is necessary for equitable participation and benefit.

Resulting from co-occurring epidemics of HIV and poverty, food insecurity is highly prevalent among people living with HIV (PLHIV) in low-resource settings^(1,2)^. Food insecurity is associated with adverse physical health outcomes among PLHIV, including challenges with antiretroviral therapy (ART) adherence, reduced CD4 count and lower life expectancy^(3,4)^. Further, food insecurity has been repeatedly associated with poor mental health outcomes, specifically depression^(3,5)^.

Previous studies have reported high rates of severe food insecurity among PLHIV in low-resource settings like the Dominican Republic (DR)^(6)^, an island nation with the highest overall HIV prevalence in the Latin American and Caribbean region, which has the second highest overall HIV prevalence globally and a high concentration of both new infections and AIDS-related deaths^(7–10)^. HIV-related deaths are among the leading causes of death in the DR, along with non-communicable diet-related diseases like heart disease, stroke and diabetes^(11–13)^. Severe food insecurity among PLHIV in the DR has been associated with increased BMI, body fat, and being categorised as overweight^(6)^, as well as detectable viral load and not having food with which to take ART, compromising both ART adherence and HIV-related health outcomes^(14)^.

Multifaceted and multisectoral food and nutrition interventions with PLHIV that integrate nutrition counselling/education and food production/agriculture in low-resource settings are needed to ameliorate food insecurity, reduce the risk of non-communicable disease and improve HIV-related health outcomes. A systematic review published in 2014 found that most food security interventions among PLHIV that have been tested are short-term food distribution programmes, and a few have tested cash assistance and livelihood programmes^(15)^. More recent studies have continued examining the effects of cash assistance and livelihood programmes^(16,17)^. Direct food assistance in low-resource settings has shown positive effects on nutritional status (i.e. primarily weight gain for underweight individuals)^(18)^, quality of life^(19)^ and household food security among PWH^(15,20)^. But food distribution programmes may have limited sustainability^(21)^, be socially unacceptable to some and may not address other factors affecting the overall health or PLHIV such as social support. There is limited evidence on the effectiveness of livelihood interventions among PLHIV^(15)^. Further, most interventions addressing food insecurity in low-resource settings have focused on individuals who are underweight^(22)^, despite the growing incidence of overweight and obesity among PLHIV^(23)^. In a study in Honduras with PLHIV who had moderate to severe food insecurity at baseline, only 11 % of participants were underweight at baseline; food assistance plus nutritional counselling led to a greater decrease in food insecurity than nutritional counselling alone, but it had the negative effect of increasing weight among those who were not underweight at baseline^(24)^.

Increasing access to both nutritious food and nutritional counselling/education are needed for PLHIV facing food insecurity and adverse HIV-related health outcomes. Pairing food-generating activities with nutrition education may contribute in multifaceted ways to improved health outcomes for low-income PLHIV facing significant individual, household and structural barriers to a healthy diet. None of the previous agricultural interventions with PLHIV have integrated comprehensive nutritional education or counselling. Nutrition education in conjunction with food assistance has been tested with PLHIV, but nutritional counselling was provided by professional nutritionists^(25)^ who are less available in low-resource settings, limiting scalability. Peer nutrition educators have been used successfully in low-resource settings^(26,27)^ but have not been tested with PLHIV, except through our own pilot studies^(28,29)^. Interventions utilising PLHIV as peer workers have successfully reduced HIV stigma and improved HIV care retention, quality and outcomes^(30–36)^, and peer workers already exist in many low-resource settings, including the DR. Peer nutritional counsellors thus represent a scalable way of implementing these services for PLHIV in such settings, and such approaches are needed to expand the provision of nutritional counselling to more PLHIV and increase social support available to PWH who may experience interrelated stigmas related to HIV, food insecurity and other marginalised identities^(37–39)^.

In sum, multifaceted, multi-sectoral and sustainable interventions in low-resource settings that increase PLHIV’s access to food and ability to optimise their dietary intake are needed for both nutritional status and improved HIV-related outcomes. This need drove the design of ProMeSA (*Proyecto para Mejorar la Seguridad Alimentaria* or Project to Improve Food Security), an NIH-funded, multi-sectoral pilot intervention that integrated urban gardening, peer nutritional counselling and nutrition education for PLHIV in the DR. Through a pilot cluster-randomised controlled trail, ProMeSA was found to have preliminary evidence of efficacy in improving undetectable viral load, ART adherence and HIV care retention^(28)^. The purpose of this paper is to explore feasibility, acceptability and implementation challenges to inform a larger intervention trial as well as other interventions designed to address food insecurity in low-resource settings.

## Methods

### ProMeSA intervention

ProMeSA integrated peer nutritional counselling, urban gardens, and garden-based nutrition and hands-on cooking workshops with the aim of reducing food insecurity and thereby improving ART adherence, HIV care retention and viral suppression. The intervention was developed after extensive formative research^(6,29,40–42)^ and involved local partners from the Dominican Ministries of Agriculture (Division of Urban Gardens) and Public Health (Department of Nutrition), CONAVIHSIDA (the National HIV/AIDS Council), and the World Food Programme (WFP). Conceptually, the intervention was based on a framework adapted from Weiser et al.^(43)^ that outlines the nutritional, psychosocial and behavioural pathways between food insecurity and HIV outcomes. Specifically, this framework shows how food insecurity can result in poor nutrition, which can lead to worse morbidity and mortality among PLHIV; food insecurity can also increase anxiety, depression and internalised stigma, which can negatively affect HIV treatment adherence (clinic visits and medication adherence)^(43)^.

Intervention components and associated activities are fully described elsewhere^(44)^. Briefly, the urban gardens component included group workshops and trainings by Ministry of Agriculture agronomists on how to establish and maintain an urban garden as well as visits to each participant’s home garden from a local agronomist to assist in day-to-day garden maintenance. Community gardens were established for participants who were unable to have gardens in their own home. The interactive, 30-min nutritional counselling sessions, provided by the clinic’s existing peer counsellor trained by WFP nutritionists, educated participants about how to include all food groups in dietary preferences, food safety and hygiene, eating healthy on a limited budget, and HIV-specific topics, such as understanding the importance of nutrition in improving ART adherence and HIV outcomes. The core curriculum included three sessions provided every other month during participants’ regularly scheduled visits to the clinic. The garden-based nutrition and hands-on cooking workshops led by WFP nutritionists applied skills of healthy and hygienic cooking learned in the peer counselling sessions and taught participants how to use their harvested produce, discussed cultural misconceptions concerning healthy eating and taught participants how to practice healthy ingredient substitution.

### Data collection

This process evaluation followed an explanatory sequential mixed methods design, quantitative data collection and analysis first, followed by qualitative data collection and analysis (the latter are used to expand upon and explain the former)^(45)^. Eligibility and recruitment of participants for the overall trial are described elsewhere^(14)^. Briefly, potential participants were approached when they visited one of two governmental HIV clinics for routine clinical appointments. Medical providers referred adult clinic patients aged 18 years or older to the study site coordinator at the clinic if they met the following criteria: registered at the clinic and had been taking ART for at least 6 months, suboptimal ART adherence (based on having missed one clinic appointment or ART refill in the past 6 months) and/or detectable viral load according to most recent screening. Those referred to the study site coordinator underwent additional eligibility screening, including whether they lived in an urban or peri-urban area in the clinic’s service area, had experienced moderate or severe household food insecurity in the past 3 months (as assessed by the Latin American and Caribbean Food Security Scale)^(46)^ and reported being able to have an urban garden at their home (physically able and having space). If eligible, the study coordinator provided an explanation of the study and conducted informed consent with those interested in participating. Quantitative data collection procedures for the intervention trial are described fully elsewhere^(28)^ and included assessments at baseline, 6 months and 12 months. All study participants provided written informed consent.

For the process evaluation, we collected additional quantitative and qualitative data. For the quantitative data, we tracked attendance of participants at intervention activities (garden and cooking workshops and nutritional counselling sessions) and included twenty-five closed-ended questions on the 12-month follow-up survey that asked about participants’ perspectives about the intervention, based on previous work^(47)^. This included fifteen questions using five-point Likert scale response options (from very unhelpful to very helpful) about how useful participants found the gardening and nutritional counselling in relation to a range of health-related outcomes (e.g. including more fruits and vegetables in their diet, ART adherence, and mental and emotional well-being; see Table 3). Another two questions asked participants about their satisfaction with the peer nutritional counselling and the cooking and nutrition workshops, using four-point Likert scales (from not satisfied at all to very satisfied). The remaining survey items asked participants about whether they were able to have a garden and the type of garden they had (e.g. home garden, community garden or both), what participants did with harvested produce, what challenges they faced and the frequency with which they prefer to receive nutritional counselling. In addition to the survey questions, objective data on attendance at garden training workshops, peer nutritional counselling sessions and cooking workshops were examined.

For the qualitative data, we conducted semi-structured interviews with a purposive subset of intervention participants (*n* 21) after their 12-month follow-up assessments to explore in-depth their perspectives and experiences with the intervention. For these interviews, we used purposive sampling, selecting individuals with co-occurring chronic non-communicable, diet-related disease (e.g. diabetes, obesity and hypertension) because of the increase in cardiovascular risks among PLHIV^(48)^ and to explore whether the intervention could be helpful for such individuals. (To determine if participants had a co-occurring condition, we used data we already had collected on individuals for our study – survey, medical chart abstraction, lab and anthropometric). To recruit a diverse study sample, an effort was made to recruit an even number of men and women. Participants were contacted by phone by the overall project coordinator after their 12-month follow-up assessments for the overall study (between October 2019 and February 2020) to see if they would be willing to participate in an additional, qualitative interview. Participants provided oral consent for this additional interview and received an additional financial incentive.

The qualitative interviews were conducted in Spanish by two trained interviewers (one as interviewer and the other as notetaker), both medical school graduates, but different from the intervention clinic site coordinator, and took 45–90 min to complete. Data were collected until thematic saturation was reached. Following each interview, interviewers took field notes about the clinic setting, thematic threads in each interview and general observations; over time, these notes help assess thematic saturation. Data saturation was determined to have occurred when no new topics or thematic trends emerged in the interviews^(49)^. The interview had a total of six sections, with each section containing between one and eight questions, and additional prompts within each question (the full interview guide has been published previously and is freely available online^(50)^). The sections included context of participants’ day-to-day lives, their physical and mental health status, participants’ experiences in accessing health services, the feasibility and acceptability of the intervention, their experiences while participating in ProMeSA (perceived impacts of the intervention), and a conclusion where participants had the opportunity to elaborate on any subject or ask interviewers any questions. Under Section 4 on the ‘feasibility and acceptability of the intervention’ (the focus for the process evaluation), the interview guide included seven questions about participants’ general impressions of the programme (e.g. what they liked and did not like), experiences and impressions of the urban garden component, challenges to starting and maintaining an urban garden, experiences and impressions with the nutritional counselling and cooking workshop, and barriers to participating in the nutritional education component of the programme.

### Data analysis

Because the current study is a process evaluation of the intervention, the results presented pertain only to the intervention clinic participants (*n* 45). For the quantitative data analysis, demographic and other quantitative data from the baseline and 12-month follow-up questionnaires are reported as measures of frequency (e.g. percentages and counts).

Regarding the qualitative data analysis, audio-recorded qualitative interviews were transcribed verbatim in Spanish by the two interviewers and an additional medical school graduate working with the team and verified. Transcripts were then uploaded to Dedoose, a web-based qualitative data analysis software^(51)^. Using field notes, interview questions and emergent themes, a codebook was developed iteratively and updated as the coding process progressed. The initial codebook was developed deductively, using general categories corresponding to the interview questions. Using field notes and following further review of the transcripts, the initial codebook was revised to inductively add emergent codes that did not fit into the code categories in the preliminary codebook. Transcripts were then coded independently by the three local interviewers/transcribers and an experienced qualitative researcher on the team, who conducted a secondary review of codes (another coding team member reviewed transcripts coded by the experienced qualitative researcher). In their analysis of transcripts and throughout the coding process, coders utilised memos and notes to make connections and thematic links across transcripts. Coding discrepancies were settled through collaborative coder consensus.

Code summaries relevant to the process evaluation component of the participant interviews were extracted from Dedoose by the lead author and used to create a participant-by-code matrix in Excel to identify overarching themes consistent across participant narratives and compare how these themes related to the quantitative data. The code matrix included general code categories that followed from the interview questions (e.g. positive characteristics of ProMeSA, negative characteristics of ProMeSA, experiences with gardening component and experiences with nutritional counselling) and any emergent codes. Under each of these code categories, memos were written for each participant based on their tagged narratives in the code summaries. Demographics for each participant were also noted in this participant-by-code matrix. The themes and subthemes that emerged from this analysis were then grouped under feasibility, acceptability and implementation challenges, as appropriate. Quotes that succinctly illustrated a trend in both the qualitative and quantitative data were selected to display this thematic alignment and provide added context and detail to the quantitative data; quotes were translated to English by the lead author, who is bilingual and Dominican, and reviewed by other bilingual co-authors (all co-authors are fluent in Spanish).

## Results

### Participant characteristics at baseline

Table 1 provides socio-demographic characteristics at baseline for the forty-five intervention participants who completed the 12-month follow-up assessments (out of a total of forty-six, one participant did not complete 12-month follow-up). Average age was 46 years (range: 25–67), 56 % were men and most (78 %) stated their nationality as Dominican, while almost a quarter identified their nationality as Haitian (22 %), 82 % reported having a primary school education or less and 18 % reported an income of less than $5000 Dominican pesos (approximately $94 USD) per month; most also reported experiencing severe food insecurity (78 %), while the remainder (22 %) were categorised as experiencing moderate food insecurity. Forty per cent of participants self-reported diagnoses of diet-related comorbid health conditions, with several participants self-reporting diagnoses of pre-diabetes, diabetes and heart problems (13 %, 11 % and 9 %, respectively). Approximately a quarter of participants also self-reported hypertension diagnoses (24 %) and high cholesterol (22 %). Further, 36 % of participants were categorised as either overweight or obese.

**Table 1:** Intervention participant (*n* 45) socio-demographics at baseline

	*n*	*%*
Age	45	
Mean	46	
sd	10·8	
Gender		
Men	25	56 %
Women	20	44 %
Nationality		
Dominican	35	78 %
Haitian	10	22 %
Education		
No schooling	8	18 %
Primary (Grades 1–8)	29	64 %
Secondary (Grades 9–12)	8	18 %
Monthly income		
0–4999 Dominican Pesos (∼0–94 USD)	8	18 %
5000–9999 Dominican Pesos (∼94–189 USD)	14	31 %
10 000–14 999 Dominican Pesos (∼189–283 USD)	10	22 %
15 000–19 999 Dominican Pesos (∼283–378 USD)	4	9 %
20 000–29 999 Dominican Pesos (∼378–567 USD)	9	20 %
Food insecurity status		
Moderate	10	22 %
Severe	35	78 %
Comorbid chronic disease		
Any	18	40 %
Pre-diabetes	6	13 %
Diabetes	5	11 %
Heart problems	4	9 %
Hypertension	11	24 %
High cholesterol	10	22 %
Overweight or obese		
Yes	16	36 %
No	29	64 %

### Feasibility

Table 2 provides the percentages of intervention participants who completed each intervention component or step (based on attendance data collected throughout the 12-month duration of the study). Overall, the ProMeSA intervention was feasible, with most participants having attended the garden workshops and been able to have a garden at home or in the community. Similarly, high percentages of participants were able to complete the three core nutritional counselling sessions and attended the cooking workshop. Below we provide details about the specific percentages completing each intervention activity, as well as quantitative data from the follow-up survey (Table 2) and related qualitative data from the interviews.

**Table 2:** Proportion of intervention participants (*n* 45) completing each programme component

Intervention step	Number of participants completing step	% of total
Nutritional counselling session 1	45	100 %
Nutritional counselling session 2	45	100 %
Nutritional counselling session 3	41	91 %
Cooking workshop	33	73 %
Urban garden workshop	38	84 %
Established an urban garden	32	71 %

#### Gardens

Most intervention clinic participants were able to participate in the gardening component of ProMeSA, with 84 % (*n* 38) attending the initial gardening workshop (Table 2) and, at the end of the study, 71 % (*n* 32) reported having been able to cultivate a garden at home, in a community garden established by the project, or a combination of these. In the qualitative interviews, participants reported planting and harvesting a wide variety of produce, including eggplant, radish, lettuce, tomatoes, beets, carrots, spinach, cilantro, okra, culantro, peppers, broccoli, maize and celery. Among those who were able to have a garden, 69 % (*n* 22) reported in the survey that they and their families consumed 50 % or more of the garden produce. In the qualitative interviews, one participant described the harvesting and tending of their garden, stating,

*To harvest, that’s beautiful, and it entertains too. You have your mind [focused on] on something. You go and take out a weed, water [the garden], see its fruit as they grow. That is something interesting.* (44-year-old man)

In addition to consuming the harvested fruits and vegetables in their own households, most participants reported sharing their harvest with other family members (88 %), neighbours (59 %) and other PLHIV (47 %). Some participants were able to sell produce in the community for added income. In fact, 75 % of participants believed that urban gardens were ‘helpful’ or ‘very helpful’ as a means to save or earn money in order to purchase other necessities (see Table 3). One participant described their harvests and how they shared them with their neighbours,

*Everything was good. I harvested like four or five times, and I would eat it all myself [or] give some to the neighbors, but I didn’t sell it. Whatever I harvested, what we grew…whether it was seven, eight…we divided it, I would take it and give to my neighbors.* (56-year-old woman)

**Table 3:** Twelve-month follow-up Likert scale survey questions on programme acceptability (*n* 45)*

Survey question	Very unhelpful/slightly helpful/not helpful or harmful	Helpful	Very helpful
In your experience, how helpful was the garden programme in:			
1. …improving your diet to be healthier overall?	(*n* 3, 6 %)	(*n* 20, 44 %)	(*n* 22, 49 %)
2. …improving your diet to include more fruits and vegetables?	(*n* 4, 9 %)	(*n* 16, 35 %)	(*n* 25, 56 %)
3. …relation to your ability to get to your doctor’s appointments?	(*n* 4, 9 %)	(*n* 17, 38 %)	(*n* 24, 53 %)
4. …relation to your ability to take your HIV medications as prescribed?	(*n* 3, 6 %)	(*n* 17, 38 %)	(*n* 25, 56 %)
5. …relation to your ability to reach or maintain your goal weight?	(*n* 3, 6 %)	(*n* 22, 49 %)	(*n* 20, 44 %)
6. …relation to your ability to save or earn money to buy things other than food?	(*n* 11, 24 %)	(*n* 15, 33 %)	(*n* 19, 42 %)
7. …relation to your mental and emotional well-being?	(*n* 2, 4 %)	(*n* 20, 44 %)	(*n* 23, 51 %)
8. …relation to your physical well-being?	(*n* 4, 9 %)	(*n* 20, 44 %)	(*n* 21, 47 %)
9. …relation to staying healthy while living with HIV?	(*n* 2, 4 %)	(*n* 19, 42 %)	(*n* 24, 53 %)
10. …relation to your ability to follow any dietary or nutritional recommendations for your health?	(*n* 1, 2 %)	(*n* 22, 49 %)	(*n* 22, 49 %)

How useful was the nutritional counselling in terms of supporting your general ability and knowledge to:			
11. …follow a healthy diet?	(*n* 2, 4 %)	(*n* 9, 20 %)	(*n* 34, 76 %)
12. …reduce unhealthy foods from your diet? (for example, foods high in fat (fried) or salt)	(*n* 5, 11 %)	(*n* 8, 18 %)	(*n* 32, 71 %)
13. …increase your intake of fruits and vegetables in your diet?	(*n* 1, 2 %)	(*n* 11, 24 %)	(*n* 33, 73 %)
14. …understand the importance of a healthy diet?	(*n* 1, 2 %)	(*n* 10, 22 %)	(*n* 34, 76 %)
15. …prepare and cook healthy meals and snacks?	(*n* 2, 4 %)	(*n* 15, 33 %)	(*n* 28, 62 %)

Survey question	More than once a month	Once a month	Less than once a month
16. How often would you prefer to receive nutritional counselling?	(*n* 10, 22 %)	(*n* 32, 71 %)	(*n* 3, 7 %)

Survey questions	Not satisfied at all/somewhat satisfied	Satisfied	Very satisfied
17. How satisfied were you with the peer nutritional counselling?	(*n* 1, 2 %)	(*n* 28, 62 %)	(*n* 16, 36 %)
18. How satisfied were you with the group cooking and nutrition workshops?	(*n* 1, 3 %)	(*n* 7, 18 %)	(*n* 30, 79 %)

Survey questions	Not well at all/not very well	Well	Very well
19. How well did the counsellor listen to you?	(*n* 2, 4 %)	(*n* 28, 62 %)	(*n* 15, 33 %)
20. How well did the counsellor understand your situation?	(*n* 2, 4 %)	(*n* 29, 64 %)	(*n* 14, 31 %)

* Percentages do not add up to 100 % due to rounding.

A minority of participants disclosed throwing produce out, and among these participants, the most common reason for discarding harvested fruits and vegetables was that they were too sick to eat them.

#### Peer nutritional counselling and cooking workshops

The peer nutritional counselling and cooking workshops were also feasible for most participants. Nearly, all participants (91 %) received all three core nutritional counselling sessions, and 73 % of participants attended the hands-on cooking workshop (Table 2). In the interviews, participants commented, especially on many things, that they learned from the cooking workshop. For example, one participant described how they learned to cook with less salt,

*Well, from the cooking workshop, what I liked the most was that I learned a lot of things…how to cook food that was healthy, because I’ll be honest, the moro [Caribbean dish with mixed rice and beans] that was made there [in the cooking workshop], I saw that it was made without sopita [bouillon cubes], and that moro was delicious. From there on out, I learned to cook with less seasoning.* (48-year-old woman)

Other participants highlighted the benefits of receiving hands-on cooking training and learning how to prepare culturally appropriate meals with healthy, fresh ingredients from the garden. One participant emphasised the importance of substituting common seasoning products such as sopita (bouillon cubes) and tomato paste containing food preservatives with fresh ingredients,

*I loved it…I tell you that what we used that day in the kitchen, I saw everything was healthy. We didn’t use any sopita [bouillon cubes] because it is harmful…I didn’t understand that sopita is harmful and also tomato paste…. [We used] natural food, very good. I loved it. I like my food more or less that way because, the more natural that you cook, the more beneficial it is for your health.* (47-year-old man)

### Acceptability

Table 3 provides responses from participants on the 12-month follow-up survey. Nearly, all participants found the different components of the intervention highly acceptable, with 93–98 % rating the gardening component as ‘helpful’ or ‘very helpful’ for following a healthier diet, helping them follow dietary recommendations, and for their mental/emotional well-being. In the interviews, one participant described the intervention’s benefits to their mental/emotional well-being,

*Ever since I joined this project, you see, my world has changed. In fact, I only leave my house to come here [to the communal garden], plant, and water my plants. My mind feels happy when I am here…it’s as if I don’t think about anything else, I just think about this [gardening].* (53-year-old man)

Many participants emphasised that comradery with other PLHIV developed through group activities helped them cope with HIV-related marginalisation. Elaborating on his previous point, one participant described the benefits of the intervention on HIV-related stress,

*Because, you know, we are learning more each day as they teach us. This program has been very beneficial, and people released the stress they had about HIV…that HIV is this, that, and the other. When we would gather— 8, 10,12 of us— to tell stories, and talk about so and so, you feel so happy.* (53-year-old man)

As another participant noted, ‘…*And they [others in the program] have given me the sense that this [HIV] is not something from another world…and you know, that helps more’ (34-year-old man)*.

Most participants also found the intervention’s urban gardening component as beneficial for HIV management (see Table 3), with 94 % rating urban gardening as ‘helpful’ or ‘very helpful’ for taking HIV medication as prescribed. Another 91 % found that urban gardens were ‘helpful’ or ‘very helpful’ to make their doctor/clinic appointments. Overall, 95 % of participants found urban gardens ‘helpful’ or ‘very helpful’ for staying healthy while living with HIV.

The nutritional counselling and hands-on, group cooking workshop were also highly acceptable to participants, and nearly all who participated were ‘satisfied’ or ‘very satisfied’ with these components of the intervention (97–98 %). Nearly, all participants (96 %) rated the nutritional counselling sessions as ‘helpful’ or ‘very helpful’ for following a healthy diet, reducing consumption of unhealthy foods (89 %), preparing and cooking healthy foods (95 %), and increasing fruit and vegetable intake (97 %). One participant emphasised the increase of fruit and vegetable consumption,

*…It has also taught me to eat a lot of things that I wasn’t eating, and that ultimately, were beneficial to my health each day.* (45-year-old man)

And even among those who were not able to have a garden, some were still able to benefit from the nutritional counselling, as described by this participant,

*But, listen, I didn’t plant, but I learned to feed myself, even if it was with someone else’s fruit…because even if it was purchased, I learned that there was a lot that I didn’t eat…that I didn’t know it was for nourishment.* (48-year-old woman)

Overall, more than 90 % of participants provided positive ratings for the peer counsellor, specifically how well the counsellor listened to them and how well the counsellor understood their situation. The nutritional counselling took place approximately every 2 months, to coincide with participants’ regularly scheduled clinic visits. However, most participants stated that they wished to receive nutritional counselling more frequently, either once a month (71 %) or more than once a month (22 %).

### Implementation challenges

Participants described several implementation challenges that impeded full participation in all programme components. For one, about 29 % (*n* 13) of participants reported at 12 months that they were unable to have a garden. The most common challenges reported by participants, regardless of whether they were able to grow an urban garden, were not having enough space (64 %), lack of protection from animals (e.g. dogs, chickens) (38 %), and no time (24 %). One participant described the challenge of space,

*…I didn’t have land [to grow food] because the land where my house is located is small. Therefore, there was no land, no way for me to sow.* (48-year-old woman)

Agronomists and other programme staff provided participants with strategies to improvise gardens in some cases, such as those in which participants had limited space. As one participant described,

*It [gardening] went very well for me. I planted, [name of peer counselor] would come to my house, [name of agronomist] also went to my house. They gave me the help I needed, they gave me fertilizer, whatever I needed to garden. I also participated here in the community garden.* (49-year-old female)

Participants also faced challenges with pests (e.g. maggots and fungi) (29 %) and not having enough support to care for the garden (27 %). One participant described the challenge concerning animals,

*But in my house, there are a lot of chickens, so we have to enclose [the garden] and [the agronomist] told me they would bring me netting. They brought me netting but the type that is plastic. I told them that because the chickens are very damaging and the dogs that I have are as well, we needed metal netting. [A home garden] wasn’t possible, but we did have a group one in [Ministry of] Agriculture’s [compound] and it was good.* (49-year-old man)

Other challenges to the gardens highlighted by participants included poor sunlight or too much shade to grow produce, poor soil, lack of transportation (e.g. to get to the garden workshops), and water scarcity. The following participant explained the challenge of not having enough sun where they live,

*Well, how do I tell you? The project of planting vegetables in the house, growing a few things…well, where I live is rented. Therefore, the patio over there…not enough sun enters from any location because there are too many plants. So, [the agronomist] tried to plant a few things over there but…it was not possible because you know if you plant something and there is no sun, it doesn’t progress.* (47-year-old man)

Participants suggested adding more seed variety, increasing the number of community gardens near participants, and more follow-up and training on gardening from the agronomists as potential strategies to improve programme implementation. Increasing the number of garden-related workshops/activities (e.g. workshops on the use of natural and industrial insecticides to protect plants) and added support to abate pests and minimise damage from local animals were also suggested as potential improvements.

Challenges to the implementation of the nutritional counselling component of the intervention included lack of transportation, limited time (e.g. due to work/family/employment obligations) and lack of availability of healthy staples (to implement what they learned in the nutritional counselling in their food preparation at home). Limited financial resources with which to purchase these items also challenged participants’ ability to practice the skills learned through nutritional counselling and in the cooking workshop. One participant discussed the challenge in applying learned skills due to limited financial resources and limited access to healthy produce in the community,

*Yes, there are moments in which I cannot apply things like [the peer counselor] [advised]. Many times, perhaps the ingredients are not available, sometimes the money is not available, too. Sometimes you have the money, and the fruits are not available…* (38-year-old man)

Another participant highlighted the challenge in getting to and from the clinic, primarily due to lack of access to transportation,

*Sometimes [I would not come to clinic], because I lived far. You know, but either way, I would come, find a ride, and come. You understand? It’s only a matter of finding a way and coming. I find a way. But sometimes it is difficult for me.* (41-year-old woman)

Participants also suggested improvements to the nutritional aspects of the intervention, including increasing the number of workshops and activities around nutrition and providing nutritional counselling at one’s home to include family in the nutritional education provided to participants (e.g. in cases where the main cook in the house was someone other than the participant).

Themes and subthemes from the qualitative data on feasibility, acceptability and implementation challenges are summarised in Table 4.

**Table 4:** Themes and subthemes in qualitative data

	Theme	Subthemes
Feasibility		
	Learned to grow and eat veggies not previously eaten Ate healthy foods and changed eating habits	Some also sold produce to supplement income; saved money on groceries; shared food with neighbours and family Avoided street food; followed principles of the ‘Pilón de Alimentación’ (food-based dietary recommendations); ate new things
Acceptability		
	Enjoyed cooking workshop and growing food Programme helped them cope with HIV-related stressors	Food cooked in class was delicious; stopped or minimised use of bouillon cubes, salt, and tomato paste Group interactions facilitated comradery; ‘my mind feels happy’ when in the garden
Implementation challenges		
	Lack of access to space/land to grow food at home Transport (and distance) to the clinic Lack of availability of healthy staples in the community and limited financial resources Other obligations prevented participation in several programme components (e.g. work, family)	Not able to take advantage of that aspect of the programme This was an added challenge to using the clinic garden Affected ability to implement principles from nutritional counselling
	Environmental conditions	Too much sun/too much shade, poor soil, pests, water scarcity
	Lack of space for growing food	Not many owned their own homes
	Inadequate number of visits from agronomists	

## Discussion

This mixed method process evaluation demonstrates that an integrated peer nutritional counselling and urban gardens programme with PLHIV experiencing food insecurity in the DR was both feasible and acceptable. This is encouraging, given that the intervention trial found preliminary efficacy of improving important HIV clinical outcomes, which, to our knowledge, represents a first for an intervention that integrated peer nutritional counselling in tandem with food generating activities with PLHIV^(28)^. Our process evaluation also identifies several implementation challenges that should be addressed to improve the benefits and reach of future iterations of the programme. High rates of food insecurity among PLHIV have been previously reported in studies in the DR, and severe food insecurity has been associated with detectable viral load as well as risk factors of diet-related non-communicable disease^(6,14)^. In addition to low rates of viral suppression among participants, nearly half also reported a diagnosis of diet-related disease, including pre-diabetes, diabetes and heart disease, diseases that like HIV are leading causes of death in the DR^(11,13)^. Thus, integrated interventions that address food production and utilisation across the range of nutritional statuses (underweight to overweight) are needed.

The ProMeSA intervention was found feasible for most participants, who were able to attend garden workshops and engage in urban gardening, receive nutritional counselling and attend a cooking workshop. Most intervention participants were able grow a diverse range of produce, which they consumed, shared with family and neighbours, and sold in the community. Selling produce not only supplemented the income of participants but also allowed financial savings that could be used to purchase other necessities. An agricultural intervention in Malawi with PLHIV who were farmers and faced food insecurity also found that agricultural yields increased participant incomes, allowing them to sell produce at markets, buy other food staples to sell at local markets, hire farm labour and address challenges with soil fertility^(52)^. In a process evaluation of a pilot agricultural and microfinance intervention with PLHIV in Kenya^(53)^, participants expressed a desire to sell produce in local markets and wished that produce grown was less perishable to do so. The ProMeSA intervention complemented food production with peer nutritional counselling and cooking workshops and participants valued learning to cook culturally appropriate meals with healthier ingredients from the garden.

ProMeSA was also found to be highly acceptable to participants, who found the components helpful for improving their diet as well as their mental and emotional well-being. The impact of the programme on coping with HIV-related stress and marginalisation through socialisation and community was notable in the narratives of participants, which is of importance considering the high levels of stigma and marginalisation experienced by PLHIV in the DR and globally^(41,54–56)^. Participants also rated the programme’s urban gardens as helpful in the management of HIV, particularly in keeping their clinic appointments and taking medications as prescribed, confirming findings from the overall evaluation, where standard measures of HIV care retention and ART adherence improved^(28)^. Participants also found the nutritional counselling component and the cooking workshop to be helpful for having a healthier diet, particularly as far as reducing consumption of unhealthy foods, increasing fruit and vegetable intake, and cooking healthy meals. For some participants, these programme components exposed them to produce that they were not eating previously. Previous programmes with PLHIV in low-resource settings found similar results, with participants reporting an increase in consumption of healthy foods prioritised in the programme^(57)^.

ProMeSA had implementation challenges that affected participation, the most notable being that 29 % of participants were unable to have a garden either at home or in the community. Several participants disclosed not having the space, and many participants did not own the homes where they lived or did not have the time to maintain a garden. Participants also described environmental challenges such as poor soil, poor sunlight, pests and water scarcity. Similar challenges were mentioned in agricultural interventions with PLHIV in Malawi^(52)^ and Kenya^(53)^. Participation in ProMeSA activities that were held away from participants’ homes, such as the nutritional counselling sessions, were hindered by lack of transportation and limited time for participation due to work and other obligations, challenges that are similar to those reported in other nutrition programmes with PLHIV in low-resource settings^(58)^. Participants also desired more seed variety, having more community gardens in closer proximity to their homes, more nutritional workshops and activities, and more training from agronomists to adequately maintain their gardens. Participants also shared that applying what they learned about nutrition and cooking was challenging due to scarcity of ingredients in the community and, in cases where such ingredients were available, having limited financial resources with which to purchase them. Despite these challenges, most participants interviewed qualitatively had a garden (81 %), were able to attend all nutritional counselling sessions and cooking workshop, and reported being able to apply the knowledge and skills they learned in the intervention. Further, the multiple components (gardens, counselling and cooking workshop) ensured that concepts were reinforced and that participants would benefit from at least some of the components. Addressing the identified challenges will be important for more equitable participation in future iterations and scale-ups of ProMeSA and similar interventions.

Given the high rates of food insecurity (60–70 %) among PLHIV in Latin America and the Caribbean^(42)^, policies and programmes that sustainably improve food security and nutrition in this population are important for optimal HIV treatment outcomes. Food insecurity and HIV have been noted as a ‘syndemic threat’, further exacerbated by the COVID-19 pandemic^(59,60)^. Addressing the underlying causes of food insecurity for PLHIV would go the furthest in ameliorating this threat; however, in the meantime, interventions such as ProMeSA will be needed. It should be noted that our intervention was sustainable because it leveraged existing human resources within the Ministry of Agriculture (agronomists) and HIV clinics (peer counsellors). Additional training of these human resources from partners at the WFP and CONAVIHSIDA build capacity around working with this population (who often possess marginalised identities) and integrating nutritional counselling. Multisectoral partnerships are needed to develop and sustain such multifaceted food security interventions.

### Limitations

This process evaluation had several limitations. First, the intervention was a pilot trial in one clinic and region; thus, results may not be transferable to other settings. Second, only participants receiving care at the clinic and prescribed ART for at least 6 months were eligible to participate in this overall study; thus, feasibility and acceptability of the intervention among PLHIV who are disconnected from care and/or ART-naïve individuals are not known. Third, social desirability bias may have influenced participant responses concerning programme satisfaction. Because programme participants received HIV-related care at the clinics where the intervention took place, it is possible that they may have been concerned about how responses may impact continuity of the intervention or their care at the intervention clinic, although this concern was addressed in the informed consent and participants were assured multiple times by interviewers that their responses would help identify ways to improve the programme. Finally, because we intended to explore the potential benefit of the ProMeSA intervention for not only HIV outcomes but also those related to CVD, we purposively sampled participants with these risks for the qualitative subsample. It is possible that, had we included a different subset of participants, they would have provided different kinds of feedback about the intervention.

### Conclusion

This process evaluation provides promising evidence that a co-located intervention integrated within HIV care in low-resource setting aiming to address food insecurity can be both feasible and acceptable to participants. As such, it contributes to the scant literature on multisectoral and multifaceted food and nutrition interventions among PLHIV facing food insecurity in low-resource settings. The mixed methods design enabled a robust analysis and provided in-depth details about participant experience in a multi-component intervention. Future process evaluations of ProMeSA could include more intervention participants in both the qualitative and quantitative components to learn about the experiences of all participants, including those with and without chronic disease. Expansion and scale-up of ProMeSA and other interventions that implement urban gardens and/or nutritional counselling should address the individual-level, structural, and environmental implementation challenges reported by participants of the pilot study and distilled through this process evaluation to ensure equitable and full participation in all programme components. Multi-sectoral interventions like ProMeSA, which improve access to healthy foods and peer nutrition education and practice, have the potential to sustainably improve food security, nutrition and quality of life of PLHIV.

## Supporting information

Celeste-Villalvir et al. supplementary material Celeste-Villalvir et al. supplementary material

## Supplementary material

For supplementary material accompanying this paper visit https://doi.org/10.1017/S1368980023002264
