# Supplementary material for: Exploring feasibility and acceptability of an integrated urban gardens and peer nutritional counselling intervention for people with HIV in the Dominican Republic
Source: Public Health Nutr. 2023 Oct 31;26(12):3134–46. doi: 10.1017/S1368980023002264 (PMC10755388; doi:10.1017/S1368980023002264)
Supplement: Celeste-Villalvir et al. supplementary material [file S1368980023002264sup001.docx]

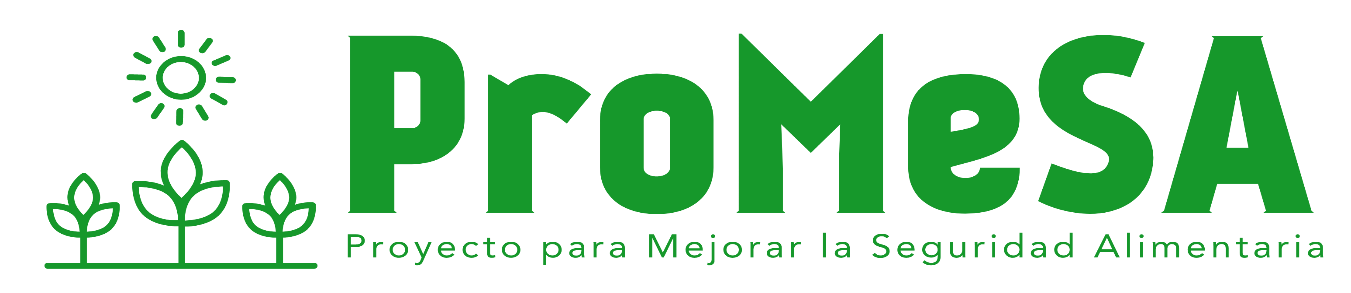


| **Semi-Structured Interview Guide Questions** | |
| --- | --- |
| **End line Interviews (12-Month Follow-up)** | |
| **Section 4. Feasibility and Acceptability af The Intervention** | |
| *Next, I would like to ask you about your opinions of the ProMeSA program.* | |
| 4.1 | Tell me more about your general impressions with the ProMeSA program (urban gardens and nutritional counseling).   - What did you like the most about the program? - What did you like the least about the program? - What would you change about the program so that it could be better? |
| 4.2 | Tell me about the experiences you had with the urban gardens in the program [Clarify if the participant had a home garden, participated in a community garden, or did both or neither].  PROBES:   - Which aspects of the garden program did you like? - Which aspects of the garden program did you not like? |
| 4.3 | What were the barriers you experienced in starting and maintaining your garden?   - How useful were the gardening classes in helping you start a garden? - How useful was the follow-up by the agronomist to your home in establishing and maintaining your garden? - What problems did you experience in having a garden (space, water, animals, diseases)? How were you able to resolve it, or not? - What were the facilitators in having a garden? What helped you? |
| 4.5 | Tell me about the experiences you had with the nutritional counseling in the program (nutritional counseling from Romelia and the cooking classes).  PROBES:   - What aspects of participating in nutritional counseling did you like? - What aspects of participating in nutritional counseling did you not like? |
| 5.6 | What were the barriers you faced in participating in nutritional education?   - What were the barriers for participating in the nutritional counseling in the SAI? - What were the barriers for participating in the cooking workshops? - How easy was it to apply your knowledge about nutrition and healthy cooking in your daily life? - What were the facilitators in helping you participate in the nutritional education and apply the lessons to your own life? |
| 4.7 | How easy or difficult was it to participate in the data collection process for ProMeSA? In other words, the survey, weight measures, height measures, blood draws, etc.?   - What changes do you suggest that we make to these measurements? - Was it difficult to come back to the clinic to get a blood draw? |
| 4.8 | Do you have other comments to improve the program for future participants? |
